# Distribution of abnormal potentials in chronic myocardial infarction using a real time magnetic resonance guided electrophysiology system

**DOI:** 10.1186/s12968-015-0133-1

**Published:** 2015-04-11

**Authors:** Samuel O Oduneye, Mihaela Pop, Mohammed Shurrab, Labonny Biswas, Venkat Ramanan, Jennifer Barry, Eugene Crystal, Graham A Wright

**Affiliations:** Medical Biophysics, University Of Toronto, Toronto, ON Canada; Imaging Research, Sunnybrook Research Institute, Toronto, ON Canada; Arrhythmia Services, Sunnybrook Health Science Centre, Toronto, ON Canada

**Keywords:** Cardiovascular magnetic resonance, Electrophysiology, Abnormal potentials, Ventricular tachycardia

## Abstract

**Background:**

Identification of viable slow conduction zones manifested by abnormal local potentials is integral to catheter ablation of ventricular tachycardia (VT) sites. The relationship between contrast patterns in cardiovascular magnetic resonance (CMR) and local electrical mapping is not well characterized. The purpose of this study was to identify regions of isolated, late and fractionated diastolic potentials in sinus rhythm and controlled-paced rhythm in post-infarct animals relative to regions detected by late gadolinium enhancement CMR (LGE-CMR).

**Methods:**

Using a real-time MR-guided electrophysiology system, electrogram (EGM) recordings were used to generate endocardial electroanatomical maps in 6 animals. LGE-CMR was also performed and tissue classification (dense infarct, gray zone and healthy myocardium) was then correlated to locations of abnormal potentials.

**Results:**

For abnormal potentials in sinus rhythm, relative occurrence was equivalent 24%, 27% and 22% in dense scar, gray zone and healthy tissue respectively (p = NS); in paced rhythm, the relative occurrence of abnormal potentials was found to be different with 30%, 42% and 21% in dense scar, gray zone and healthy myocardium respectively (p = 0.001). For location of potentials, in the paced case, the relative frequency of abnormal EGMs was 19.9%, 65.4% and 14.7% in the entry, central pathway and exit respectively (p = 0.05), putative regions being defined by activation times.

**Conclusions:**

Our data suggests that gray zone quantified by LGE-CMR exhibits abnormal potentials more frequently than in healthy tissue or dense infarct when right ventricular apex pacing is used.

## Background

Ventricular tachycardia (VT) associated with chronic myocardial infarction are the leading cause of sudden cardiac death [1]. To guide ablation therapy, conventional electrophysiology (EP) substrate mapping delineates the myocardial infarct area using voltage mapping, then uses pace-mapping to define the VT exit as well as local electrogram (EGM) characteristics to identify areas with slow conduction [2]. In the majority of VT cases arising after the patient has suffered a myocardial infarction, slow conduction regions are part of the re-entry circuit in the arrhythmogenic substrate [1], often detected with the voltage maps as well. These regions of the re-entrant path have been found to contain diastolic potentials [3]. These abnormal local electrograms (EGMs) originate from the propagation across surviving viable myocardium separated by fibrosis in a zig-zag activation pattern [4,5]. The combined local conduction delays produce abnormal signals with multiple signal components, each representing the nonsynchronous depolarization of the different bundles of the surviving myocardial fibers. These abnormal potentials, recorded during electroanatomical mapping, are classified as fractionated potentials or as isolated/late potentials. Diastolic and fractionated potentials are pathognomonic potentials for VT substrate and their locations are the effective targets for ablative therapy [6].

Abnormal potentials have been analyzed in several studies. Hsia et al. [1,7] compared the correlation between late potentials and arrhythmogenic substrate of reentrant VT circuits and found that, out of the 133 sites recorded, a higher prevalence of late potentials were recorded near the isthmus (89%) compared to entrance (57%) or exit (20%). Similarly, comparable studies have identified higher prevalence of late potentials at sites classified as central or proximal in the reentry circuit as compared to other sites (exit or outer loop sites) [8,9]. Abnormal potentials were shown not only to correlate with the reentry pathway but also to have direct correspondence to successful ablation targets. When ablation strategies have focused on targeting isolated potentials the results have proven to be effective [4,5,10]. Furthermore, other studies have also indicated that abnormal potentials can be used to identify critical isthmuses with signals recorded during sinus rhythm (SR) [6,11,12], paced-controlled rhythm [6] or both [10,13].

Therefore, we aimed to: 1) identify regions of isolated/late and fractionated potentials in sinus rhythm and controlled-paced rhythm in post-infarct animals; and 2) to characterize the relative prevalence of these abnormal potentials in critical sites and areas labeled as gray zone as quantified by late gadolinium enhancement cardiovascular magnetic resonance (LGE–CMR).

## Methods

### Animal preparation

Six pigs weighing an average of 35 kg were used for the study. The chronic infarct model was obtained for each animal by undergoing infarct induction and four weeks recovery; after approximately the four weeks, an MR-guided EP procedure was conducted. Before each procedure, animals were placed under general anesthesia induced with an intramuscular injection of ketamine (30 mg/kg) and atropine (0.05 mg/kg), and maintained by inhalation of 1-5% isoflurane. Also lidocaine (30 ml of 2% lidocaine in 250 ml of saline) and 50 mg/ml amiodarone, were administered during the procedure; anticoagulation with intravenous heparin (70 units/kg) was also administered. Then, the animals were intubated and mechanically ventilated (20–25 breaths/minute). For infarct induction, complete coronary occlusion was achieved distal to the second diagonal branch of the left anterior descending artery (LAD) for 90 minutes or 60 minutes by inflation of a percutaneous balloon dilation catheter (Sprinter Legend Balloon Catheter, Medtronic, Minneapolis, MN), and was followed by reperfusion. The different occlusion times change the extent and heterogeneity of the infarct.

For the EP procedure, incisions of femoral vessels, carotid artery and/or jugular vein were performed and access secured with 9 F introducer sheaths for the purpose of administering medications and inserting mapping catheters. Finally, ECG leads I, II, and III were monitored throughout the infarct induction procedure as well as the electrophysiology procedure to track animal health. The animal care committee at Sunnybrook Research Institute approved this protocol and all the procedures were conducted following institutional guidelines.

### Imaging protocol and image processing

**Figure 1:**
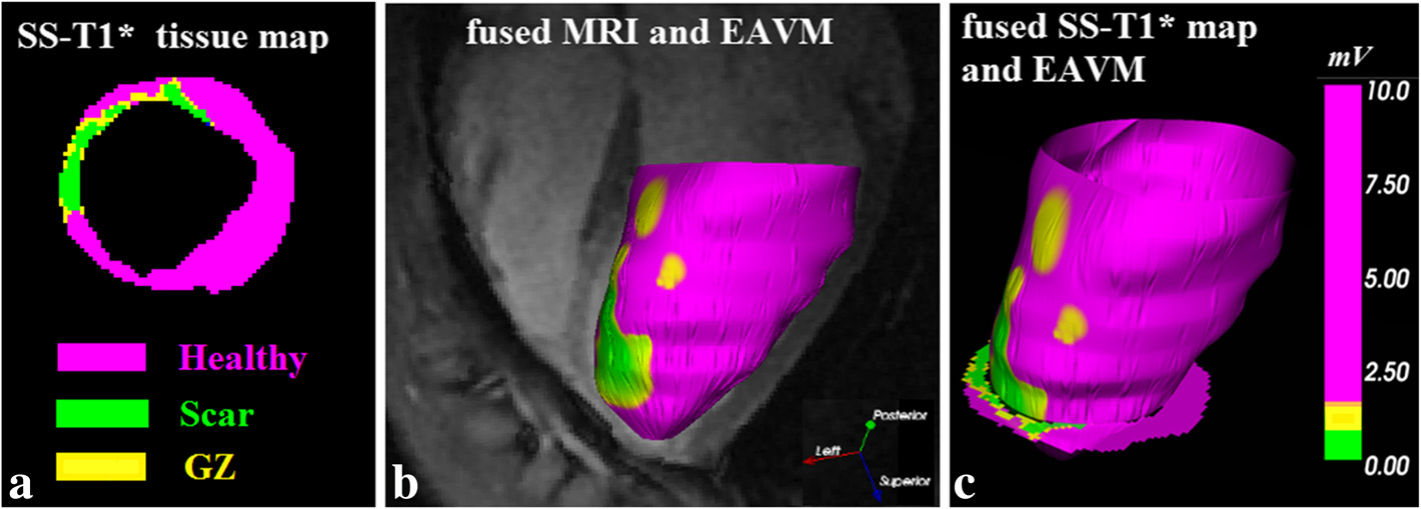
**Fusion of multi-contrast late enhancement information and voltage signal of electroanatomical map. (a)** LGE images using the MCLE sequence (or steady-state T1*) were processed using a fuzzy clustering algorithm to classify each pixel as infarct (green), healthy myocardium (purple), or gray zone (yellow). **(b)** SSFP-derived meshes were overlaid with voltage amplitude information obtained from the MR-Guided endocardial mapping procedure, resulting in an EAVM (color coded as per color bar). **(c)** After the procedure, EP meshes were fused with the MCLE-derived images.

A 1.5 T GE Signa (GE Healthcare, Milwaukee, USA) with a 5-inch surface coil was used to acquire anatomical scans. The left and right ventricle were fully imaged in long axis and short axis directions, yielding multiple 2D MR images using a respiratory gated sequence. The principal anatomical scans were performed with SSFP-CINE with the following parameters: TE/TR = 1.1/3.6 ms, FA = 45°, BW = 62.5 kHz, NEX = 1, and views per segment (vps) = 12, slice thickness = 5 mm, FOV = 210 mm (phase FOV 0.8 or 0.9), Nx = 224, Ny = 160. This resulted in an approximate in-plane resolution of 0.9 × 1.2 mm. The cine SSFP sequence produced 20 phases over the heart cycle, acquired on average over 10–15 second breath-holds. Following the anatomical scans, we performed LGE imaging using a multi contrast late enhancement (MCLE) sequence recently developed in our lab [14]. MCLE was shown to yield more reliable and reproducible measures of the infarct core and gray zones than conventional techniques. This sequence produces images with multiple contrasts where infarct can be visualized as an area of fast T1* recovery (T1* is the apparent T1 relaxation, shorter than the true T1 due to the continuous SSFP readout). The MR scans started 5 minutes after injection of 0.44 mmol/kg of Gd-DTPA (Magnevist, Berlex Inc., Wayne, NJ). The parameters for the MCLE sequence were: TR/TE = 2.7/1.3 ms, readout FA = 45°, BW = ±125 kHz, VPS = 16, FOV = 210 mm, slice thickness = 5 mm, 192 × 160 (phase FOV 0.8 or 0.9) imaging matrix and NEX = 1. An inversion pulse was placed such that the infarct-enhanced images from the continuous SSFP acquisition are acquired during diastole. The resulting images from the MCLE sequence were used to classify the different myocardial tissues states. Six to eight images were utilized from the diastolic cardiac phase to extract the signal recovery rate (1/T1*) and the steady-state plateau of the recovery curve signal for each pixel within the (left ventricle) LV. After obtaining T1* and steady-state values for each pixel, we used a fuzzy C-means algorithm to automatically classify each pixel as infarct, healthy myocardium, gray zone, or blood, as previously described in [14]. We segmented the infarct core, gray zone and healthy myocardium in each of our MCLE short-axis images (see Figure 1a). The classification results were processed and imported into our electrophysiology visualization system as an endocardial mesh (Figure 1c).

### Real-time MR-guided electrophysiology system

The MR-guided EP apparatus was comprised of 8.5Fr MR-compatible Vision™ catheters, (Imricor Medical Systems, Burnsville, USA) and a prototype Bridge™ EP Recording System (Imricor Medical Systems, Burnsville, MN); we used a 4-electrode catheter with 2 mm spacing between the first two distal electrodes, 3 mm spacing between second and third electrode and 1 mm spacing between third and fourth electrode. Bipolar recordings were made with the distal electrode pair (1 and 2). The catheters were placed in the (right ventricle) RV or LV using active catheter tracking with MR-guidance as reported previously [15]. The catheters were advanced under full MR guidance with a 3D LV shell created from segmentation of SSFP-cine images acquired in the same session, with the animal resting in the same position; this eliminated the need for additional registration or alignment. The LGE images were loaded into the visualization software Vurtigo (vurtigo.ca) to directly identify the navigation targets, (i.e., the infarcted areas) (see Figure 1). At each point of contact, the intracardiac signals and the three-dimensional spatial coordinates were recorded. As detailed in our previous study we calculated spatial error associated with fusion of EAM surface and EP points by measuring the average perpendicular distance between EP points and mesh surface [15].

### Electrophysiology procedure

**Figure 2:**
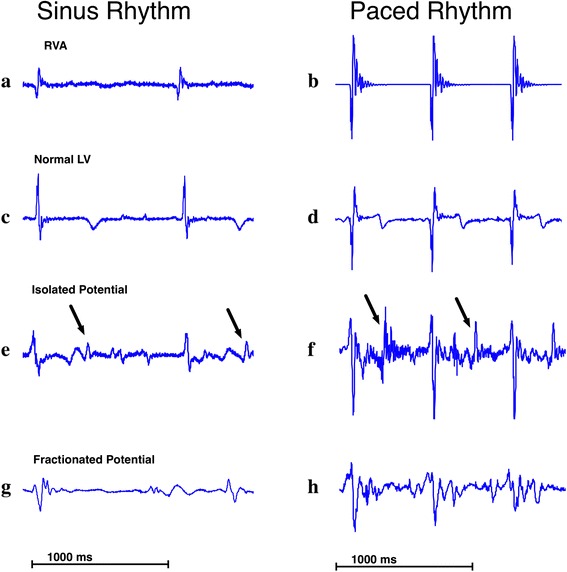
**Examples of abnormal EGMs recordings from animals with myocardial infarction.** Shown are the EGM recordings in RV and LV in the sinus **(a, c, e, g)** and paced **(b, d, f, h)** condition. Tracings **(a)** and **(b)** show RVA signals, c and d represent normal healthy myocardium recordings. The following recordings are all acquired in non-healthy tissue. In **(e)** and **(f)** the local EGM recorded by the mapping catheter displays an isolated potential (oblique arrows) that is separated from the ventricular EGM by an isoelectric line of at least 20 ms. Finally, **(g)** and **(h)** are the recorded EGM fractionated and the width is > 102 ms; (RVA = Right ventricular apex, LV = left ventricle).

Using the MR-guided EP system, we conducted substrate mapping during sinus rhythm for each animal, right after the imaging acquisition. The EP mapping data was collected in the following sequence: 1) a voltage map was constructed for the endocardial surface of the left ventricle under real-time MR guidance;

2) Right ventricular apex (RVA) electrical stimulation was then performed, with a second catheter advanced into the right ventricle to reduce overlap of action potential signals commonly seen in the porcine model. For the latter, performing pacing at a rate faster than the animal’s normal sinus rhythm (on average 400 msec cycling) and increasing the current until muscle capture was assured, the natural pacemaker was overridden. Abnormal potentials were defined in the following manner: 1) distinct bipolar electrogram recordings inscribed after the end of the RVA reference signal (R-peak), separated from the major initial component of the local ventricular electrogram with an isoelectric interval greater than 20 ms [11] in both sinus and paced cases; and 2) fractionated potentials as multicomponent signals of long duration >133 ms [8] and greater than underlying signal noise (0.1 mV) in both sinus and paced rhythms. The former method is an internationally accepted method from Bogun et al. [11], while the later, as described in the above-reference, is more recent. The remaining EGMs displaying both above-mentioned characteristics were also defined as abnormal. Only endocardial sites with bipolar electrogram recordings acquired during sinus or right ventricular pacing were analyzed for abnormal EGMs (see Figure 2). All EGMs recordings within the LV EP map were analyzed for the presence or absence of abnormal potentials. EGMs with high electrical noise, or poor signal quality reducing accurate assessment of abnormal EGMs were discarded. Signal definition and rejection at this time it was conducted visually and manually, with an experienced observer.

To estimate the degree of local activation time delay in the LV, an isoelectric interval was measured as the timing interval from the onset of the second upstroke in the ventricular electrogram (verified by the corresponding RVA reference signal) to the earliest component of the abnormal EGM. The incidence of abnormal EGMs in different infarct regions as defined by voltage amplitudes and by LGE-CMR was determined. Regions of low voltage potentials were classified as those with voltage amplitude < 1.5 mV, specifically < 0.5 mV for dense scar and 0.5 mV-1.5 mV for gray zone [2]. Critical central pathways were defined as regions of late (or delayed) activation time in infarct or gray zone tissue and it was hypothesized to support VT, in a similar manner as the isthmus of the reentry circuit.

### Statistical analysis

Categorical variables were compared using a chi-squared test. The test allowed to detect group differences using frequency (count) data. To determine the statistical significance of the frequency of potentials in for different tissue classifications, the recorded (or observed) potentials and expected abnormal potentials were analyzed; where the expected potentials corresponded to the normalized distribution.To determine the statistical significance of the frequency of potentials in the different presumed locations/regions of the slow conduction path (entry, exit and critical central pathway), we compared observed and expected proportion with a chi-square test (http://graphpad.com/quickcalcs/chisquared1/ and http://www.quantpsy.org/chisq/chisq.htm). Continuous variables were compared using one-way ANOVA test (http://danielsoper.com/statcalc3/). A value of p < 0.05 was considered statistically significant. MATLAB (The Mathworks, Natick, MA) was used for statistical analyses.

## Results

### Electrophysiology mapping

In our studies during the electrophysiology mapping, we recorded a total of 3945 good quality EGMs from 6 animals. EGMs were recorded in both SR (2469 points) and paced rhythm (1476 points). During SR the signals recorded resulted in 609 low voltage EGMs and 558 abnormal EGMs; for the paced rhythm, 308 and 360 EGMs were founds to be low voltage and abnormal respectively. Also, our analysis focused on the relationship between abnormal potentials and tissue properties as classified by MCLE. Tissue classification results were processed and imported into Vurtigo as an endocardial mesh. Abnormal potentials recorded during the mapping procedure were assigned to locations on the tissue classification mesh using a closest point algorithm; this process resulted in EGM characteristic to tissue property pairings for various regions.

**Figure 3:**
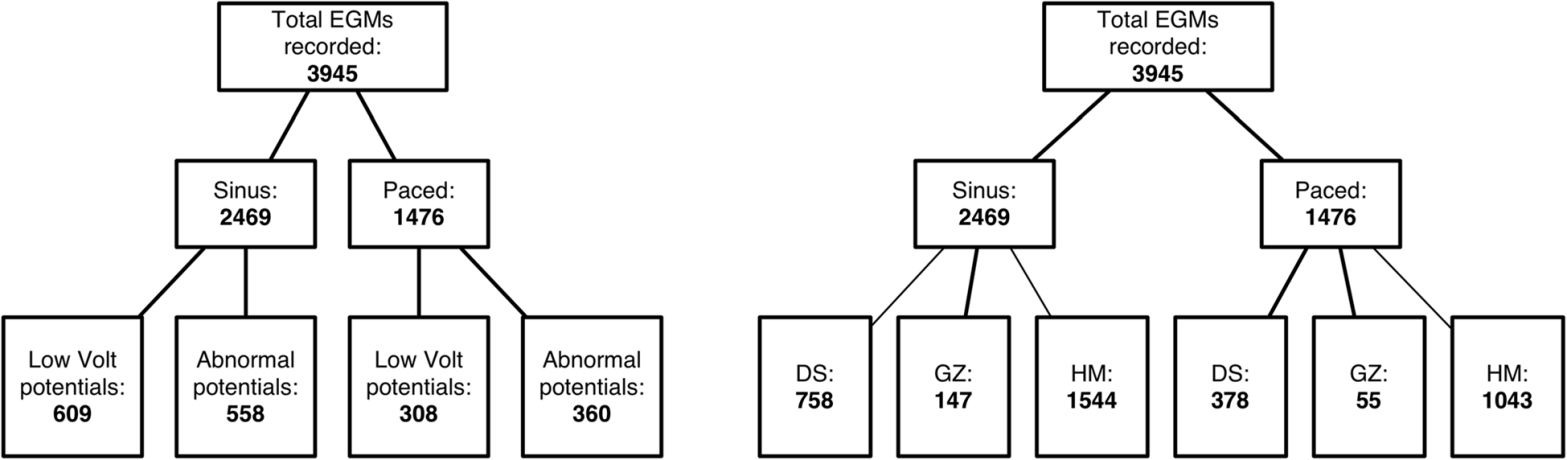
**Distribution of abnormal potential recordings in sinus and paced rhythm for low voltage potentials and abnormal potentials (left); and within scar, gray zone and healthy myocardium regions (right) of the endocardium.** Low Volt = low voltage, DS = dense scar, GZ = gray zone, HM = healthy myocardium.

Out of the 2469 points in SR and 1476 in paced rhythm points, we recorded 758, 147 and 1544 EGMs from MR-defined regions of dense infarct, gray zone and healthy myocardium respectively in the SR case. For the paced case we recorded 378, 55 and 1043 EGMs for MR-defined dense infarct, gray zone and healthy myocardium respectively. Results are summarized in Figure 3. Finally, the average spatial error due to cardiac and respiratory motion of the acquired mapping points was calculated to be 2.1 ± 1.1 mm.

### Distribution of abnormal potential signals in different cardiac rhythms

Sites classified as dense scar, gray zone, and healthy myocardium by MRI within the low voltage and abnormal potentials groups were further examined. Relative prevalence of abnormal EGMs in a given region was calculated as the ratio of EGMs classified as low voltage or abnormal over the total number of EGMs recorded in scar, gray zone and healthy myocardium, respectively. In general, our analysis determined that low-voltage potentials had a higher relative prevalence in dense scar in sinus, whereas abnormal potentials had a higher relative percentage in gray zone regions in paced rhythm. Specifically, for low-voltage in sinus rhythm, relative prevalence was 28%, 26% and 22% in dense scar, gray zone and healthy myocardium respectively (p = 0.01 among the three groups). For low-voltage during pacing the relative prevalence of low-voltage potentials was 26%, 4% and 20% for dense scar, gray zone and healthy myocardium respectively (p < 0.001). Conversely, for abnormal potentials in sinus rhythm, relative prevalence was equivalent for all EGM measurements in the mapped regions of interest, and calculated to be 24%, 27% and 22% (p = 0.3) in dense scar, gray zone and healthy tissue respectively; in the paced rhythm case the relative prevalence of abnormal EGMs was 30%, 42% and 21% (p = 0.001 between each group) in dense scar, gray zone and healthy myocardium respectively. These results are summarized in Table 1. Finally, our results indicate that in paced rhythm gray zone has higher frequency of abnormal potentials with respect to healthy myocardium (p < 0.01) and statistically trending towards higher frequency with respect to dense scar (p < 0.1).

**Table 1:** Distribution of abnormal potential EGMS recorded in scar, gray zone and healthy regions

**Animals N = 6**	**Sinus rhythm**				**Paced rhythm**			
	**Dense scar**	**Gray zone**	**Healthy myo**	**p-value**	**Dense scar**	**Gray zone**	**Healthy myo**	**p-value**
Total EGMs recorded	758	147	1544		378	55	1043	
Low voltage potentials	214 (28%)	38 (26%)	337 (22%)	p = 0.01	98 (26%)	2(4%)	208 (20%)	p = 0.01
Abnormal potentials	183 (24%)	39 (27%)	336 (22%)	p = 0.3	113 (30%)	23(42%)	224 (21%)	p = 0.001

Values in parentheses indicate fraction of low voltage or abnormal potentials as a percentage of total EGMs in scar, gray zone or myocardium. EGMs are shown in rhythm and vs. paced rhythm. p-values are calculated as the difference between the tissue groups.

### Distribution of abnormal potential signals in different locations

**Figure 4:**
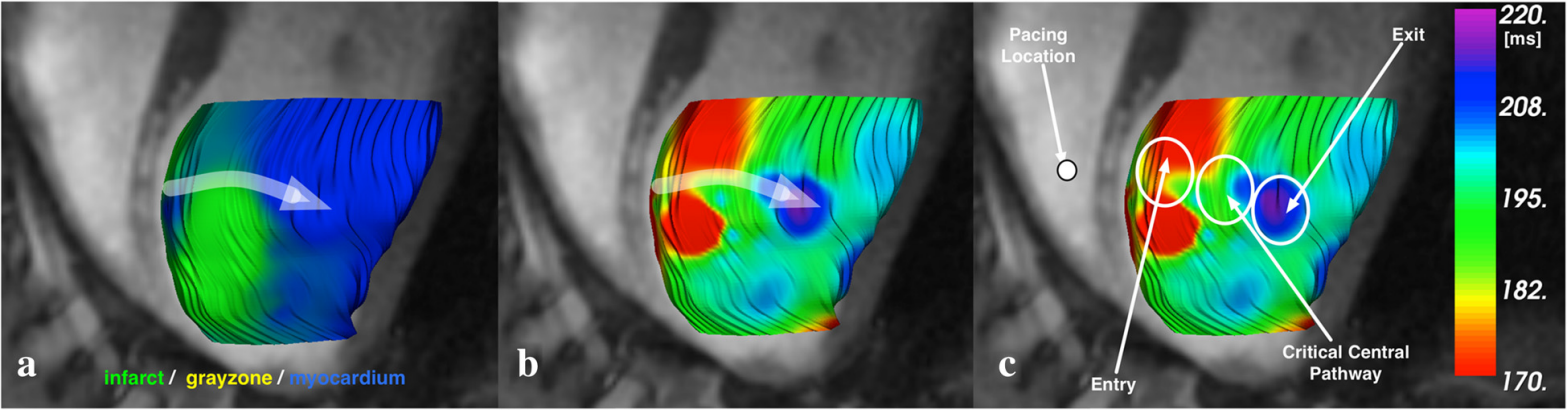
**Activation map recorded during mapping procedure.** The color index reveals the continuity between the earliest and latest activated areas typical of later activation time capable of supporting VT. The presumed depolarization wavefront is drawn to indicate potential pathway. **a)** classification of tissue **b)** map of time of depolarization. Arrow shows presumed slow conduction path; **c)** designation of regions in presumed slow conduction path.

**Figure 5:**
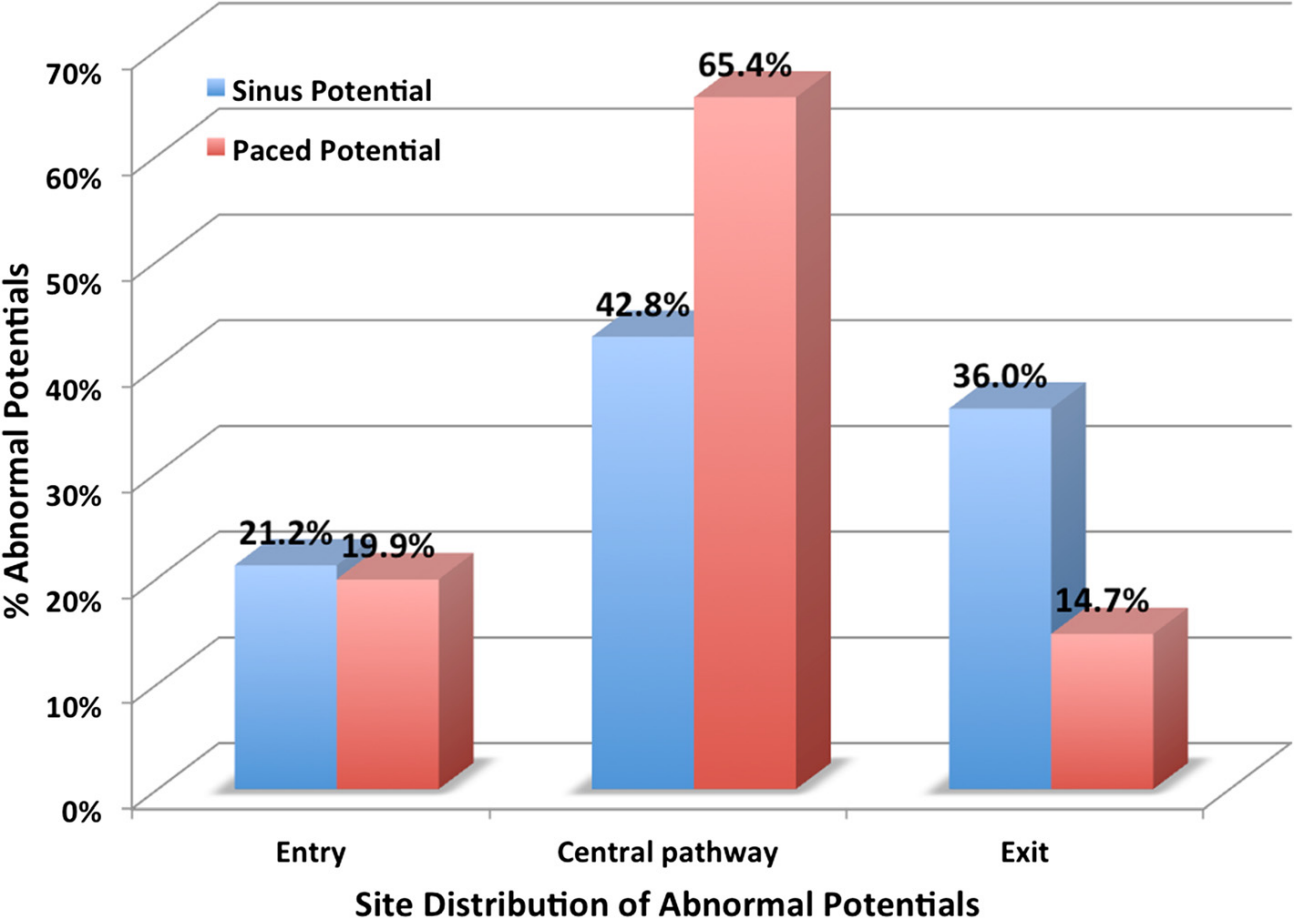
**Distributions of abnormal potentials.** Entry, critical central pathway and exit are shown in the figure. The distribution of abnormal potentials is based on Figure 2. The critical central pathway location is associated with the presence of abnormal potentials. Using a comparison of proportion test, we determined that the frequencies between each distribution were statistically significant p < 0.05.

Distributions of abnormal potentials across locations in assumed target regions as a function of rhythm were also analyzed. The target regions of the presumed slow conduction path (entry, critical central pathway and exit, see Figure 4) were inferred from depolarization times of local activation/arrival signals relative to all activation times. Local activation times for recorded points classified as dense infarct and gray zone were subdivided into quartiles for each animal (note, health myocardium was not included): 1) the first quartile of the earliest activation times within MR-determined regions of dense infarct or gray zone were classified as being associated with the entry; 2) 2^nd^ and 3^rd^ quartile were classified as within the critical central pathway; and 3) latest activation times (4^th^ quartile) are considered to be within the exit region. The choice of quartile regions was made to favour repeatability of calculations and analysis. Only relative activation times in the dense infarct and gray zone were considered, to reduce any bias due to the presence of health myocardium or excessive distance from pacing site (pacing case). Therefore, the distribution of all measured potentials across entry, critical central pathway and exit will be, by definition, 25%-50%-25% respectively. Considering only the abnormal potentials observed in these regions we asked if their spatial distribution was different than that for all potentials. Our results show a signficant and relative difference between the sinus rhythm case and paced case. Specifically, our results show that, in the sinus case, there is an unexpectedly high relative fraction at the exit, specifically 21.2%, 42.8%, and 36.0% of the abnormal potentials in the regions were seen in the entry, central pathway, and exit regions respectively; in the paced case, the abnormal EGMs were more likely to be seen in the central pathway region, with distribution of 19.9%, 65.4% and 14.7% in the entry, critical central pathway and exit regions respectively (as shown in Figure 5). Using a comparison of proportion test, we determined that each of these distributions differed from the expected distribution of 25:50:25 in a statistically significant way (p < 0.05). The significance of these findings is presented in the next section.

## Discussions

### Abnormal potential signals distribution

The main findings of this study are that: (1) abnormal potentials are more prevalent in the MR-defined gray zone and (2) this relationship is more evident during RVA pacing. CMR determined gray zone has been shown to correlate with all-cause post-MI mortality and with inducibility for VT for patients with myocardial infarct scars; therefore it would be important to characterize the prevalence of abnormal potentials in areas labeled as gray zone as quantified by LGE–CMR. Our data suggest that abnormal potentials are more prevalent in the MR-defined gray zone, particularly during pacing. This is a novel finding, and provides additional insights into the analysis of RVA stimulation; It is believed that overlapping activation fronts may limit the identification of multiple components during sinus rhythm, where overlapping refers to the activation of various parts of the myocardium due to non-uniform activation of the endocardium generated by the Purkinje fibers as well as to beat-to-beat heart rhythm variability in sinus rhythm. This limitation can be mitigated by simplifying the evaluation to one propagation front; specifically, RVA pacing from a known location creates a dominant action potential wavefront that can identify abnormal potentials otherwise not detected [6]. Our results are consistent with this notion as RVA pacing suppresses overlapping signals and reduces the influence of adrenergic and cholinergic nerve fibers present on the swine endocardium. The heterogeneous nature of gray zone with bundles of surviving myocytes responsible for the irregular propagation of the action potential may well explain our observation. Also, our results are in line with previous studies that hypothesized that overlapping of EGMs or a particular orientation of a line of block with respect to the activation front may preclude the identification of multiple components during SR [6]. This limitation can be overcome by changing the activation front, such as during RVA pacing, to identify otherwise undetected abnormal EGMs. This is important because the authors of the previous study also claim that these EGMs could better identify VT-related slow conduction areas relative to low-amplitude electrograms [6]. Further, we noticed that in both sinus and paced rhythm there was a percentage of low volume abnormal potentials in healthy tissue. Based on visual inspection we noticed that such points were recorded recorded in healthy myocardium, which was located adjacent to the infarct region. In addition, a few points of low voltage in healthy myocardium were also acquired very close or touching the valve, while others probably did not have a very good contact with the ventricular.

Our results showes a lower prevalence of low voltage points in gray zone than health myocardium in the paced rhythm case; Although gray zone in that case showed a lower prevalence of low voltage points, the average signal amplitude was lower than in the healthy myocardium. So while the average signal in the healthy myocardium was above 1.5 mV, there were several low voltage points. We also found that, in the paced case, a higher relative percentage of abnormal signal potentials was associated with sites in the presumed slow central pathway compared to entry or exit, based on activation activation times. These findings are in line with findings of Nakahara *et al.* [13], where a higher concentration of abnormal EGMs (late potentials) was found in the putative isthmus and also with Hsia et al. [7], where the majority of these delayed and fractionated endocardial electrogram recordings were found in critical central pathway sites. In this study we did not report nor conduct a rigorous correlation study between low-voltage and MR-defined dense scar, since it was the focus of our previous publication [15].

Abnormal potentials are more associated with the location of the VT isthmus than low amplitude potentials [6,7,11,16]. Locations of VT-related conducting channels have been shown to be only grossly estimated by low peak bipolar voltages (<1.5 mV) measured during SR without tachycardia induction. Also, recently it has been shown that the presence of isolated potentials inside the voltage channel significantly increases the specificity for identifying the clinical VT isthmus [17]. Furthermore, linear radiofrequency ablation lesions designed to transect these channels of slow conduction have been shown to be on average longer in length by several centimeters when the isthmus has not been effectively identified and only voltage maps are known (e.g. in hemodynamically unstable patients) [18]. In contrast, abnormal EGMs reflecting anisotropic late activation times can identify VT-related slow conduction regions. Specifically, sites with isolated/late potentials and fractionated potentials during sinus/paced rhythm reflect late local activation of bundles of healthy myocytes that enable conduction and become critical components of a reentry circuit. Abnormal potentials acquired during endocardial mapping have been shown to correspond to appropriate ablation targets with greater specificity than simple peak voltage mapping [10,19].

Identifying abnormal potentials becomes especially important in cases where the patient has hemodynamically unstable VT, or non-inducible VT; otherwise, in these patients, electrophysiologists only have the information from the voltage maps, which as noted above, have low specificity for VT substrate identification [2,6]. Point-by-point mapping of the LV is time consuming and leads to prolonged procedure/anesthesia time, and the low specificity of this technique requires additional diagnostic procedures [6]. The point-by-point mapping also has limitations related to catheter-tissue contact and largely unspecified spatial resolution. In our previous study [15] we showed that CMR information used in electrophysiology studies can accurately predict areas of myocardial fibrosis identified with bipolar voltage mapping. We believe that a strategy for identification of sites for mapping and ablation is achievable when using CMR combined with abnormal electrical signal information; this added information is desirable to due to its greater specificity compared to peak voltage mapping alone. More specifically, gray zone volume with LGE-CMR has been shown to correlate with all-cause post-MI mortality and with inducibility for ventricular tachycardia (VT) [20,21].

## Conclusions

Our data suggests that gray zone quantified by LGE-CMR has high correspondence to regions with a high proportion of abnormal potentials. This correspondence is higher when right ventricular apex stimulation is employed. Abnormal electrograms are commonly observed in sinus rhythm and paced rhythm from and in close proximity to slow central pathways in other studies. Future investigation into the clinical outcome associated with ablation of VT using gray zone information and signal morphology is warranted

## Abbreviations

LGE-CMR: Late gadolinium enhanced cardiovascular magnetic resonance
LV: Left ventricle
RV: Right ventricle
RVA: Right ventricular apex
VT: Ventricular tachycardia
EGM: Electrogram
EP: Electrophysiology
SR: Sinus rhythm
MCLE: Multi contrast late enhancement
